# Managing the transition from paediatric to adult care for HIV, Kenya

**DOI:** 10.2471/BLT.19.232702

**Published:** 2019-09-13

**Authors:** Irene Njuguna, Kristin Beima-Sofie, Caren Mburu, Cyrus Mugo, Danae A Black, Jillian Neary, Janet Itindi, Alvin Onyango, Jennifer Slyker, Laura Oyiengo, Grace John-Stewart, Dalton Wamalwa

**Affiliations:** a Kenyatta National Hospital, Research and Programs, P.O. Box 20723-00202, Nairobi, Kenya.; b Department of Global Health, University of Washington, Seattle, United States of America (USA).; c Department of Pediatrics and Child Health, University of Nairobi, Nairobi, Kenya.; d Department of Epidemiology, University of Washington, Seattle, USA.; e Kenya Medical Research Institute, Kisumu, Kenya.; f National AIDS and STI Control Program, Ministry of Health, Nairobi, Kenya.

## Abstract

Expansion of access to diagnosis and treatment for human immunodeficiency virus (HIV) and a high incidence of HIV infection in adolescence has resulted in a growing population of adolescents and young adults living with HIV. The prevalence of poor retention in care, insufficient viral suppression and loss to follow-up are higher among adolescents and young adults compared with other age groups. Poor outcomes could be attributed to psychosocial changes during adolescence, but also to poor transitional care from paediatric to adult HIV services. In many countries, transition processes remain poorly defined and unstructured, which may jeopardize treatment adherence and retention. We describe existing definitions of transition and transition frameworks, and key elements of transition as proposed by key national stakeholders in Kenya. Our consensus definition of transition is “a planned process by which adolescents and young adults living with HIV, and their caregivers, are empowered with knowledge and skills to enable them to independently manage their health.” Transition should begin soon after disclosure of HIV status until an adolescent gains the necessary knowledge and skills and is willing to move to adult services, or by 25 years of age. Proposed key elements of transition are: target ages for milestone achievement; readiness assessment; caregiver involvement and communication with adult clinics; flexibility to return to adolescent or paediatric clinics; group transition; and considerations for adolescents with special needs. Retention in care, linkage to care and viral suppression are important markers of transition success. Proposed definitions and key elements could provide a framework for structuring transition programmes in other countries.

## Résumé

L'accès élargi au diagnostic et au traitement du virus de l'immunodéficience humaine (VIH) et l'incidence élevée de l'infection par le VIH à l'adolescence ont entraîné une hausse de la population d'adolescents et de jeunes adultes atteints du VIH. La prévalence de mauvais continuums de soins, de suppressions virales insuffisantes et d'interruptions du suivi est plus élevée chez les adolescents et les jeunes adultes que dans d'autres groupes d'âge. Ces mauvais résultats peuvent être attribués aux changements psychosociaux qui interviennent à l'adolescence, mais aussi à la médiocrité de la transition entre les services de soins pédiatriques et adultes du VIH. En Afrique subsaharienne, les processus de transition sont mal définis et peu structurés, ce qui peut nuire au respect et à la poursuite des traitements. Nous décrivons ici les définitions existantes de la transition et des cadres de transition, ainsi que les éléments clés de la transition proposés par les principales parties prenantes nationales du Kenya. Notre définition consensuelle de la transition est: « processus planifié qui permet aux adolescents et aux jeunes adultes atteints du VIH, ainsi qu'à leurs aidants, de disposer des connaissances et compétences nécessaires pour gérer leur santé de manière autonome ». La transition devrait débuter peu après la communication de la séropositivité et durer jusqu'à ce que l'adolescent ait acquis les connaissances et compétences nécessaires et qu’il souhaite passer à des services pour adultes, ou jusqu’à l'âge de 25 ans. Les éléments clés proposés de la transition sont: âges cibles pour le franchissement de certaines étapes; évaluation de la maturité; implication des aidants et communication avec les services pour adultes; possibilité de revenir à des services pédiatriques ou pour adolescents; transition de groupe; prise en compte des adolescents ayant des besoins spéciaux. Le continuum de soins, le lien entre les soins et la suppression virale sont d'importants marqueurs de la réussite de la transition. Les définitions et éléments clés proposés peuvent offrir un cadre pour structurer les programmes de transition dans d’autres pays.

## Resumen

La ampliación del acceso al diagnóstico y tratamiento del virus de la inmunodeficiencia humana (VIH) y la alta incidencia de la infección por el VIH en la adolescencia han dado lugar a una población creciente de adolescentes y adultos jóvenes que viven con el VIH. La prevalencia de una retención inadecuada en la atención, la supresión viral insuficiente y la pérdida de seguimiento son mayores entre los adolescentes y los adultos jóvenes en comparación con otros grupos de edad. Los resultados negativos pueden atribuirse a cambios psicosociales durante la adolescencia, pero también a una atención de transición deficiente de los servicios pediátricos a los servicios para adultos con VIH. En África subsahariana, los procesos de transición siguen siendo mal definidos y desestructurados, lo que puede suponer un riesgo para el cumplimiento y la retención del tratamiento. Describimos las definiciones existentes de transición y los marcos de transición, así como los elementos clave de la transición propuestos por las principales partes interesadas a nivel nacional en Kenia. Nuestra definición consensuada de transición es “un proceso planificado mediante el cual los adolescentes y adultos jóvenes que viven con el VIH, y sus cuidadores, son capacitados con conocimientos y habilidades que les permiten manejar su salud de manera independiente”. La transición debe comenzar poco después de la revelación del estado serológico respecto al VIH hasta que el adolescente adquiera el conocimiento y las habilidades necesarias y esté dispuesto a trasladarse a los servicios para adultos, o a la edad de 25 años. Los elementos clave de la transición propuestos son: edades objetivo para el logro de los hitos; evaluación de la preparación; participación de los cuidadores y comunicación con las clínicas para adultos; flexibilidad para regresar a las clínicas para adolescentes o pediátricas; transición de grupos; y consideraciones para los adolescentes con necesidades especiales. La retención en la atención, la vinculación a la atención y la supresión viral son marcadores importantes del éxito de la transición. Las definiciones propuestas y los elementos clave podrían proporcionar un marco para estructurar los programas de transición en otros países.

## ملخص

إن التقدم في التوصل إلى تشخيص، والحصول على علاج لفيروس نقص المناعة البشرية (HIV)، وكذلك ارتفاع نسبة الإصابة بهذا الفيروس في مرحلة المراهقة، إلى تزايد عدد السكان من المراهقين والشباب المصابين بفيروس نقص المناعة البشرية. إن انتشار سوء البقاء في الرعاية ومكافحة الفيروس غير الكافية والافتقار إلى المتابعةـ يظهر بشكل أعلى بين المراهقين وشباب البالغين، مقارنة بالفئات العمرية الأخرى. يمكن أن تعزى النتائج الضعيفة إلى التغيرات النفسية والاجتماعية خلال فترة المراهقة، ولكن أيضًا إلى سوء الرعاية الانتقالية في خدمات فيروس نقص المناعية البشرية من الأطفال إلى البالغين. في جنوب الصحراء الكبرى الأفريقية، تظل العمليات الانتقالية غير واضحة وغير واضحة المعالم، وهو ما قد يعرض الالتزام بالعلاج والاحتفاظ به للخطر. نحن نصف التعريفات القائمة للانتقال وأطر الانتقال، والعناصر الرئيسية للانتقال، وفقاً لما اقترحه أصحاب المصلحة الوطنيون الرئيسيون في كينيا. كان تعريفنا بالإجماع لعملية الانتقال هو "عملية مخطط لها تعمل على دعم المراهقين وشباب البالغين المصابين بفيروس نقص المناعة البشرية ومقدمي الرعاية، بالمعرفة والمهارات اللازمة لتمكينهم من إدارة صحتهم بشكل مستقل". يجب أن يبدأ الانتقال على الفور بعد الكشف عن حالة الإصابة بفيروس نقص المناعة البشرية، حتى يكتسب المراهق المعرفة والمهارات اللازمة، ويرغب في الانتقال إلى خدمات البالغين، أو عند سن 25 عامًا. العناصر الرئيسية المقترحة للانتقال هي: الأعمار المستهدفة لتحقيق الإنجاز؛ وتقييم الجاهزية، ومشاركة مقدمي الرعاية وتواصلهم مع عيادات البالغين؛ والمرونة في العودة إلى عيادات المراهقين أو الأطفال؛ والانتقال الجماعي؛ واعتبارات للمراهقين ذوي الاحتياجات الخاصة. يعد البقاء في الرعاية، والارتباط بالرعاية، ومكافحة الفيروس، هي مكونات مهمة لنجاح عملية الانتقال. يمكن أن توفر التعريفات المقترحة والعناصر الأساسية إطارًا لهيكلة برامج الانتقال في البلدان الأخرى.

## 摘要

人类免疫缺陷病毒 (HIV) 诊断与治疗途径的扩大以及青春期 HIV 感染的高发病率导致越来越多的青少年和年轻人被确诊为 HIV 感染者。与其他年龄组相比,青少年和年轻人中普遍存在护理保留率低、病毒抑制不足和失访率较高的现象。结果不良可归因于青春期在社会环境影响下的个人心理变化,也可归因于从儿科过渡到成人 HIV 服务的转诊护理不良。在撒哈拉以南非洲地区,过渡进程仍然定义不明确且结构不合理,这可能会影响治疗依从性和保留率。我们描述了现有的过渡和过渡框架定义,以及肯尼亚主要国家利益相关者所提出的过渡的关键因素。我们达成共识的过渡定义是“感染艾滋病毒的青少年和年轻人及其护理者通过一个有计划的过程获得知识和技能,从而具备保持身体健康的自主能力。”在披露 HIV 状况之后不久就应开始过渡,直到青少年获得必要的知识和技能并愿意转向成人服务,或者年满 25 岁。现已提出的过渡关键因素为:完成主要阶段性成就的目标年龄;准备程度评估;护理者参与以及与成人诊所的沟通;重返青少年或儿科诊所的灵活性;团体过渡;以及针对有特殊需求的青少年的考量因素。护理保留率、护理联动和病毒抑制是过渡成功的重要标志。对于撒哈拉以南非洲地区和其他资源匮乏地区而言,提出的定义和关键因素能够为在其他国家构建过渡计划提供一个框架。

## Резюме

Расширение доступности диагностики и лечения вируса иммунодефицита человека (ВИЧ) и высокая заболеваемость ВИЧ-инфекцией в подростковом возрасте привели к увеличению количества людей молодого возраста и подростков, живущих с ВИЧ. По сравнению с другими возрастными группами у подростков и молодежи чаще встречается несоблюдение назначенного режима лечения, недостаточная супрессия вирусной нагрузки и неявка для последующего наблюдения. Неблагоприятные исходы лечения могут быть связаны с психосоциальными переменами, характерными для подросткового возраста, а также с плохим качеством медицинского обслуживания при переходе от педиатрической схемы лечения ВИЧ к взрослой. В странах Африки, расположенных к югу от Сахары, процесс такого перехода остается плохо определенным и бессистемным, что может угрожать соблюдению назначенного режима лечения и удержанию пациентов. Авторы описывают существующие определения перехода и его рамки, а также ключевые элементы перехода, предложенные основными национальными партнерами в Кении. Общепринятое определение перехода звучит так: «Это спланированный процесс, в ходе которого подростки и молодежь, живущие с ВИЧ, а также лица, обеспечивающие им уход, получают знания и навыки, которые позволяют им заботиться о здоровье без посторонней помощи». Переход должен начинаться вскоре после предоставления информации о ВИЧ-статусе и продолжаться до тех пор, пока подросток не получит необходимые навыки и знания и не выразит желание перейти на взрослую схему лечения, или до достижения пациентом возраста 25 лет. Были предложены следующие ключевые элементы переходного процесса: деление на целевые возрастные группы для достижения определенных промежуточных этапов; оценка готовности; привлечение лица, осуществляющего уход, и общение с представителями клиник для взрослых; гибкая возможность возвращения в клинику для детей или подростков; групповой переход и учет особенностей подростков с особыми потребностями. Обеспечение непрерывности лечения, привязка к уходу и супрессия вирусной нагрузки — вот важные маркеры успешного перехода. Предложенные определения и ключевые элементы могут служить в качестве основных принципов для структурирования программ перехода и в других странах.

## Introduction

Expansion of paediatric antiretroviral therapy (ART) and treatment services has improved survival among children living with human immunodeficiency virus (HIV). However, globally, HIV-associated mortality among adolescents 15–19 years of age remains high.^1^ In addition, adherence to medication is suboptimal^2^ and loss to follow-up among 15–24-year-olds is twice as high as that of younger adolescents (10–14 years of age) or adults (25 years of age or older).^3^ Transition from paediatric or adolescent HIV care to adult services is emerging as a critical step for maintaining good outcomes among adolescents and young adults.

Structured transition programmes can support adolescents and young adults living with HIV to successfully transition into adulthood. In high-income countries, transition from paediatric to adult care services usually involves a change of clinic and /or providers, largely because care is provided as a specialized service and transitional models of care are available.^4^^–^^6^ The lack of structured transition programmes reflects a broader gap in bridging from paediatric- and adolescent- to adult-oriented services for chronic illness in general, and for HIV care specifically.^7^ The model of care in sub-Saharan Africa is predominantly non-specialist, comprising integrated clinics where adults, adolescents and children living with HIV are seen by the same staff in the same place.^7^^,^^8^ However, adolescent-friendly services, usually in the form of a dedicated adolescent clinic day, are increasingly being implemented.^7^^,^^8^ Although specific services that address the unique needs of adolescents may be available, practices remain varied.

The World Health Organization (WHO) defines HIV care transition as purposeful planned movement from paediatric models of care to adult services, the goal of which is to support adolescents gain life skills to independently manage their care.^9^ However, at a programmatic level, definitions and tracking systems for transition are varied^8^ and transition-focused services are short-lived, typically occurring in the context of research projects.^7^

In this article, we describe current definitions of transition, existing transition frameworks and propose transition definitions and key components of transition programmes that could be used in programmatic settings. We outline our team’s experience engaging a broad range of stakeholders, as part of the Adolescent Transition to Adult HIV Care for HIV-infected Adolescents in Kenya study, which has the goal of developing and testing a comprehensive transition package of care. We also describe limitations that may hamper implementation of transition programmes.

## Transition definitions

Common definitions of transition in HIV care are based on multiple criteria including: age; completion of a formal transition process; awareness of HIV diagnosis; HIV knowledge; attendance at an adolescent transition clinic; being clinically stable; being pregnant or married; and demonstrating independence.

### Age-based definitions

Age-based definitions may be used alone or in combination with other criteria that defines transition. Three distinct ways of defining the age of transition can be identified in the research literature: (i) by developmental stage; (ii) by achievement of independence; and (iii) by programme categorization. 

The first definition is based on expectations of stages in development when the shift to adulthood occurs. Transition programmes are largely designed to prepare 18–25-year-old adolescents for transfer to adult care.^7^^,^^10^^–^^13^ This range coincides with the ages at which adolescents are becoming independent. Although the age to begin the process of transition may be defined, the Adolescent Trials Network (an American-based network of HIV clinics participating in adolescent HIV research) has emphasized that adolescents need to have attained sufficient maturity to navigate adult services.^12^ Even among adolescents with developmental delay, age is still an important determinant of defining who is prepared for transition. One study reported high numbers of transition failures when transfer to adult care was done at 21 years of age and researchers proposed moving the definition of age of completing transfer to 25 years of age.^10^

The second approach to defining age cut-off for transition is based on society’s expectations of a specific age when individuals achieve independence. Common ages that have been used to define transition include 12, 15 and 18 years, but a wider range exists (from 12 to 22 years of age).^7^^,^^14^

The third approach to defining age cut-off for transition is based on an individual attaining the age of re-categorization in data systems within HIV care programmes.^8^ Almost a third (57) of 218 health-care facilities across sub-Saharan Africa lack a working definition of adolescents and 80% (174 out of 218) do not disaggregate treatment outcomes by age.^15^ Common programmatic age cut-offs for transition are 12, 15 and 18 years of age. These definitions may result in changes in access to certain services, for example access to youth clinics and support groups,^14^ or changes in how medical records are handled or stored.

WHO guidelines describe transition in integrated clinics as “adoption of different approaches of care recognizing the child’s changing ability and needs.”^9^ For adolescents in integrated clinics, transition may mean coming to the clinic on their own, making their own clinic appointments and being able to pick their own medications. However, these variables are rarely captured. In the International Epidemiology Databases to Evaluate AIDS South African collaboration, where clinics are largely integrated, researchers compared pre- and post-transition retention in care and viral suppression levels using different age thresholds as proxies for when transition might occur.^16^ However, although convenient and perhaps the only available approach to analyses of transition given data limitations, using age definitions does not take into account developmental challenges or the family and social circumstances, which adolescents face and that could appropriately influence development and transition.

### Formal processes

Formal HIV care transition processes are common in high-income countries. Adolescents reach a certain age and then go through a formal transition plan to ensure specific goals are attained. A formal readiness assessment tool^10^ or an informal process is used to document readiness to transition. A few programmes in low- to middle-income countries have used readiness assessment tools. The Baylor Kalogo programme in Zimbabwe included two to three readiness assessment questions administered at each clinic visit and training modules on transition in peer support groups. Adolescents were required to meet specific criteria before transfer to adult clinics.^7^ The Thailand Happy Teen 1 programme included a multidisciplinary team providing individualized and group-based transition training at clinic visits.^17^ In some HIV programmes, training lasting from 90 minutes to 2 days and 6-week transition camps have been conducted.^7^^,^^18^^,^^19^

Youth transition clinics to support transition are sometimes used.^7^ These clinics are usually in the form of adolescent clinic days, in which a day of the week is dedicated to adolescent care. Activities may include regular clinic care with additional peer-support activities, or only peer-support activities. Ideally, adolescent clinics would fully engage adolescents and have well-trained health-care workers.^18^ However, there is evidence of underutilization of these clinics,^20^ with only 32% of 379 Ugandan adolescents reported to be regular attenders in one study. In addition, many of these programmes lack policies, guidelines and tools, staff and community support, resulting in poor support for and utilization by adolescents.^21^^,^^22^

### Other definitions of transition

In Nigeria, individuals’ awareness of their HIV status and HIV knowledge were used to define transition.^8^ However, assessment was informal and did not specify what would be regarded as enough knowledge. Including knowledge of HIV status in the transition definition may explain some of the early transition ages reported in sub-Saharan Africa,^7^ as disclosure of HIV status tends to occur in early adolescence.

In Nigeria, pregnancy and marriage are used as criteria for transition of adolescents to adult care.^8^ Similarly, in Kenya, pregnancy in adolescent girls results in abrupt transfer from paediatric or adolescent clinics to maternal and child health units^23^ and these girls may not return to the adolescent clinic after delivery. Adolescent pregnancy, early marriage and HIV infection may be markers of additional vulnerabilities and may result in worse outcomes.^24^^–^^26^

Health-care workers have also described transition as demonstrating independence, either by adolescents attending clinics on their own or collecting their own medication,^27^ but this has not been systematically assessed.

In specialized paediatric HIV clinics, particularly in South Africa, transition is defined by reaching12 years of age and being stable on treatment.^28^

## Transition models of care

Two distinct groups of models of adolescent care exist. The first model involves a physical transfer, whereby adolescents transition from specialist paediatric or adolescent services to adult services with a change in clinic or provider, or both. In the second model, adolescents remain within comprehensive services and both clinic and provider remain the same. For this second group of adolescents, transition definitions are difficult to envision. Adolescent-friendly clinics, may offer some definitions. For example, adolescents who transition from an adolescent clinic day to an adult clinic day may be considered to have transitioned.^7^^,^^27^ However, poor utilization of adolescent clinics remains a problem.^20^

## Transition frameworks 

Transition frameworks from resource-rich settings may be adapted for use in low-resource settings. The Got Transition tool^29^ developed in the United States of America provides a framework for transition services and a transition timeline beginning at 12 years of age and ending at age 26 years that can be adapted by clinics. The tool includes 6 core elements: (i) establishing a transition policy; (ii) tracking transition progress; (iii) assessing transition readiness; (iv) planning for adult care; (v) transferring to adult care; and (vi) integration into an adult practice. The first three core elements may apply to any model of care or setting. 

In 2012 and 2014^30^ the United States Agency for International Development released a HIV care transition guide for use in sub-Saharan Africa that includes four of the Got Transition core elements (transition tracking and monitoring; transition readiness; transition planning; and transfer to adult care). However, where the tool was piloted, health-care providers did not fully use the tool due to time challenges and tool complexity.^18^ How best to simplify transition tools and motivate providers to use them remain important questions. The International Center for *AIDS*Care and Treatment Programs at Columbia University, New York, United States, has also published transition guidelines that incorporate HIV care transition readiness, planning, transfer and transfer completion.^31^ However, data on utilization and outcomes from the use of these tools are lacking. At a national level, among the five countries with the highest burden of adolescent HIV infection, only the Kenya national guidelines for ART comprehensively address transition, providing age-based transition milestones and a standard data collection tool to act as a reminder to health-care workers to discuss transition during visits.^32^

## Successful transition

Successful transition has previously been described either qualitatively by assessing adolescent experiences after transition or quantitatively by assessing post-transition outcomes including CD4+ T-lymphocyte count, viral load, mortality, adherence to therapy and retention in care.^17^^,^^33^^–^^37^ Many of these studies were conducted in resource-rich settings, where there is actual transfer to adult services. In sub-Saharan Africa, few studies have assessed transition readiness and validated tools are lacking. Researchers have developed and validated a transition readiness assessment for adolescents with chronic health-care needs in the United States.^38^ While the tool can be used across multiple illnesses, these tools would need to be adapted for the different medical care context in sub-Saharan Africa settings.

## Kenyan experience

Kenya has a generalized HIV epidemic, with over 1.5 million people living with HIV in 2018.^39^ Of these, an estimated 105 200 (7%) are children younger than 15 years of age and 184 718 are aged 15–24 years of age.^39^ In 2018, There were 8000 new infections among children younger than14 years of age.^39^ While the Kenya Ministry of Health guidelines for HIV care recognize transition as a key element of successful HIV care,^32^ there is little guidance on definitions of transition or how to measure success.

The Adolescent Transition to Adult HIV Care for HIV-infected Adolescents in Kenya study is a cluster-randomized trial aimed at addressing gaps in transitional care for adolescents and young adults 10–24 years of age who are living with HIV and attending public HIV care clinics in Kenya. The study aims to (i) understand current transition practices; (ii) develop a transition tool for use in programmatic settings in Kenya; and (iii) test the developed transition tool in a cluster randomized trial. To begin national discussions on transition, the study team has partnered with the Kenya health ministry, HIV care partners, county HIV care directors, implementing partners, mental health experts, health-care providers from paediatric and adult settings, and adolescent representatives. 

The first phase of the study included assessment of clinic transition practices and outcomes in a nationally representative sample of 102 HIV clinics representing over 12 000 adolescents and young adults living with HIV. For the second phase, we reviewed existing transition definitions, and adapted and developed transition definitions and frameworks for use in programmatic settings in Kenya. The third phase, the cluster-randomized trial (NCT number: 03574129), is planned to begin in October 2019. The trial will test the effectiveness of an adolescent transition package of services to improve transition readiness, retention in care and viral suppression among adolescents and young adults living with HIV in clinics randomized to receive the intervention.

### Proposed transition definition

**Fig. 1:**
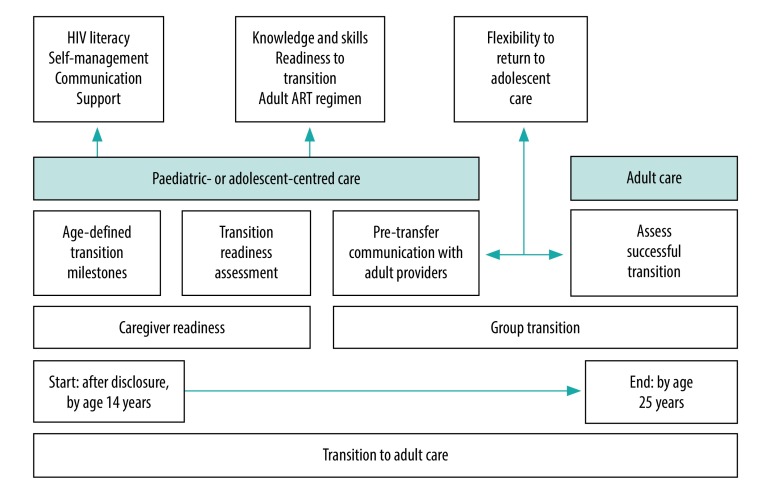
Overview of a proposed framework for successful transition of children and adolescents to adult HIV care

ART: antiretroviral therapy; HIV: human immunodeficiency virus.

Our consensus definition of transition is “a planned process by which HIV-infected adolescents and young adults, and their caregivers, are empowered with knowledge and skills to enable them to independently manage their health.” The transition process is proposed to begin soon after disclosure of HIV status and not later than 14 years of age. Transition preparation would continue until adolescents gain the knowledge and skills they need, and are ready and willing to move to adult-oriented services (Fig. 1). Transition should be completed by 25 years of age. The currently recommended age at which transition discussions should begin is much earlier than previously documented in the literature from high-income countries,^10^ which may allow the desired psychological preparation before transition.^23^^,^^40^ Beginning transition preparation earlier may be particularly beneficial in sub-Saharan Africa, as adolescents often present to clinics alone, due to challenges such as transport costs, being orphans, caregiver illness, and not having disclosed their status to others, however, may not necessarily have the knowledge and skills to successfully manage their care.^41^

### Proposed transition framework

For the HIV care transition processes in Kenya, we have proposed the following core elements: transition milestone achievement in four key areas (HIV treatment literacy; self-management; communication skills; and support); transition readiness assessment; caregiver involvement; communication with adult clinics where necessary; flexibility to return to adolescent clinics; group transition where possible; and special considerations for adolescents with special needs (Fig. 1; Box 1). Other clinical considerations include patients being on an optimized ART regimen and clinically stable (virally suppressed and no active opportunistic infections). In clinic settings where adolescent and adult services are offered separately, communication between providers, group transition (referring to transition of adolescent cohorts who have been in care together) and transition support groups may be key.

Box 1 **Summary of key elements and definitions of successful transition of children and adolescents to adult HIV care**

**Transition dates**

Transition starts soon after disclosure of HIV status, preferably from age 14 years. Transition ends by age 25 years, but with consideration of the individual’s developmental milestones.

**Readiness assessment**

Adolescents are assessed by a health-care worker from 17 years of age for:

• transition knowledge and skill milestones; (HIV literacy; self-management; communication and support);

• optimized regimen of antiretroviral therapy (on an oral adult regimen);

• stable on treatment (no new opportunistic infections or active infections);

• holistic approach (addressing school, social and family concerns);

• willingness to transition.

**Caregiver readiness**

Caregivers are willing to allow adolescents to take responsibility for their own health.

**Communication with adult clinic**

Adult clinic staff are aware of the adolescents being transitioned to their clinic.

**Flexibility to return to adolescent services**

At 18–25 years of age adolescents can receive services in adolescent clinics if they are uncomfortable in adult clinics.

**Group transition**

Group transition and support groups are available where possible.

**Special considerations**

Transition process can be slower or faster for pregnant adolescents, those with delayed or early milestones and adolescents with disability.

**Definition of successful transition**

Successful transition includes the following:

• linkage to care in adult clinic (at least one clinic visit within 3 months of their last clinic visit in an adolescent or paediatric care system);

• retention in care (three or more visits over a 12-month period);

• viral suppression (consistent viral load measurements below the level of detection for ≥ 12 months).

If a clinic is integrated (adult, adolescent and paediatric) successful transition would include retention and viral suppression as above occurring after 25 years of age or from the time of achievement of key transition milestones.

HIV: human immunodeficiency virus.Notes: Our consensus definition of transition was developed as part of the Adolescent Transition To Adult HIV Care for HIV-infected Adolescents in Kenya study, based on a workshop conducted on 17–18 September 2018 with 32 key stakeholders purposively selected from a broad range of HIV care leaders.

### Proposed definition of successful transition

In Kenya, we proposed to define successful transition of individuals as follows: (i) at least one clinic visit within 3 months of attending an adult clinic, or the time at which it is documented that they have reached the knowledge and skill milestones to navigate autonomous care, or 25 years of age, whichever is first (linkage); (ii) three or more visits over a 12-month period (retention); and (iii) consistent viral load measurements below the level of detection for ≥ 12 months (viral suppression). Post-transition follow-up is recommended to include contact at 3 months to evaluate linkage to care and at 6–12 months to assess retention, viral suppression and experiences in adult care. These definitions align with existing guidelines for clinic visits recommended to be at least every 3 months for adolescents and young adults living with HIV. In programmatic settings, retention and viral suppression are routinely tracked. In programmes where adult, adolescent and paediatric clinics are integrated, and documentation of reaching knowledge and skills milestones is available, achievement of milestones will be used to define the transition time-point. If the information is not available, 25 years of age will be used. Retention and viral suppression following this time or age will be used to approximate to post-transition outcomes. Therefore, our proposed definition of successful transition fits well into existing programmes, but will require modification to report age-disaggregated outcomes.

## Benefits of transition programmes

Transition programmes provide a structured approach for adolescents and young adults living with HIV to gain the necessary knowledge and skills they need for their care in adulthood and to gain confidence as they enter adult services. Adult care systems in many countries have often been described as judgemental, depersonalized and overburdened, and adolescents have reported feeling afraid, unprepared and alone in adult care.^14^^,^^42^^,^^43^ As a result of the differences in health-care culture in adult and paediatric systems, health-care workers have expressed difficulty letting-go of adolescents and young adults living with HIV whom they have often looked after since infancy or childhood.^14^^,^^42^^,^^44^ Lack of effective communication between adult and paediatric providers is also seen as a barrier to effective transition programmes.^42^ In clinics where providers do not change, adolescents and young adults living with HIV may face fewer barriers to transition, but few data are available on the benefits of these systems. However, qualitative evidence suggests that transition, if well planned, could be a positive event.^34^^,^^40^ In addition, while adult clinics may be unwelcoming, they may foster independence and are better placed to address some of the biological age-related realities of care in young adults. Data on transition experiences is lacking and more research in this area is needed.

## Limitations of transition programmes

Where HIV transition programmes have been found to be effective, a key component of these programmes has been having a dedicated transition coordinator or team^13^ who are responsible for identifying adolescents in need of transition, and for tracking and follow-up after transition. An effective transition programme may require re-organization of staffing to meet these additional roles. However, many health-care systems are overburdened and clinicians may not have enough time to address transition needs. Innovative models, such as group transition programmes, peer-led programmes or programmes and tools that empower caregivers to support transition, may be feasible options. The Zimbabwe Zvandiri programme is perhaps the most successful peer-led programme.^45^ Zvandiri is heavily invested in community adolescent treatment experts; these are 18–24-year-old HIV-infected adolescents who provide support for adolescents who have not attained treatment goals. The strength of the programme seems to lie largely in strong community and health ministry partnerships and collaborations.

Tools for tracking transition are also generally lacking. The Kenya health ministry introduced a standard tool for health-care workers to remind them to address key adolescent needs during clinic visits and a visual poster placed in the clinic that reminds health-care workers what transition milestones to assess by age.^46^ Transition preparation is one of the items included in the tool. Tracking individuals’ progress, however, remains challenging. Development of standardized transition tools, such as transition booklets, tracking tools, procedures for assessing successful transition and support systems remain important, as does an understanding of the specific resources needed to support their use. Whether these tools improve transition readiness, translate to better health outcomes or are cost–effective remains unclear.

## Conclusion

Our definitions and key elements of transition developed in Kenya could be translated to other settings with similar models of care. While identifying a practical way to track and assess transition in programme settings remains challenging, we provide a framework for better understanding transition definitions and processes that can support the development of interventions to improve transition outcomes.
